# A bibliometric analysis of COVID-19 and physical activity

**DOI:** 10.1097/MD.0000000000030779

**Published:** 2022-09-30

**Authors:** Yuting Zhang, Mengtong Chen, Chunlong Liu, Zhijie Zhang, Xihua Fu

**Affiliations:** a Clinical Medical College of Acupuncture Moxibustion and Rehabilitation, Guangzhou University of Chinese Medicine, Guangzhou, China; b Luoyang Orthopedics Hospital of Henan Province, Luoyang, China; c Department of Infectious Diseases Unit, Panyu Central Hospital, Guangzhou, China.

**Keywords:** bibliometric analysis, CiteSpace, Corona Virus Disease 2019, physical activity, VOSviewer

## Abstract

Due to the huge impact of coronavirus disease 2019 (COVID-19) on a global scale, the level of physical activity during confinement has become a widespread concern. This study summarizes the development of performance and research trends in COVID-19 and physical activity over the last 3 years.

**Methods:**

Research publications on COVID-19 and physical activity in the past 3 years were downloaded from the Web of Science database. CiteSpace and VOSviewer software were used to analyze the authors, published outputs, journals, cited authors, countries and institutions, co-cited journals, cited references, and keywords. Statistical and centrality analyses were used to identify the active authors, core journals, basic references, hot topics, and cutting-edge fields.

**Results:**

A total of 1331 papers was retrieved. SMITH L was a prolific author in the field of exercise intervention in COVID-19 with 11 publications. *International Journal of Environmental Research and Public Health* was the most productive journal (179 publications) and the most cited journal (1324). The most productive countries and institutions in this field were the USA (322 publications) and Harvard Medical School (21 publications). The four hot keywords in COVID-19 and physical activity research were physical activity, exercise, health, and mental health.

**Conclusions:**

This study provides researchers with directions to intervene in changing levels of physical activity during the COVID-19 pandemic and valuable information for researchers in the field of sports medicine to identify potential collaborators, collaborating institutions, hot issues, and research frontiers.

## 1. Introduction

Coronavirus disease 2019 (COVID-19) is an infectious disease caused by the SARS-CoV-2 virus. In December 2019, the first case of COVID-19 was reported in the South China Seafood Market in Wuhan, Hubei Province, China. The World Health Organization (WHO) declared COVID-19 a public health emergency of international concern (PHEIC) on January 30, 2020.^[1]^ On March 12, 2020, the World Health Organization declared the COVID-19 outbreak a global pandemic.^[2]^ The coronavirus pandemic has been wreaking havoc across the globe, straining health facilities in many countries, and increasing the number of infections and deaths every day. According to official statistics from the WHO, on February 14, 2022, there were 1,570,734 new confirmed cases and 6417 new deaths on a single day.^[3]^ Countries are taking preventive measures to contain the spread of the virus, reduce the overall infection and mortality rates, and improve public health.

The WHO has provided specific guidelines, including wearing surgical masks, keeping people apart, and isolating infected or close contacts as requested by the government.^[4]^ By maintaining the necessary social distancing and enforcing stricter segregation policies, people were making fewer unnecessary outings and staying at home longer. These measures reduced the amount of time individuals spent in physical activity, which had adverse effects on all systems and organs, and these effects were more pronounced and difficult to reverse in patients with chronic diseases and the elderly.^[5]^ Most people infected with coronavirus are the elderly, those with underlying diseases, those lacking exercise, and those with poor living habits. Studies have shown that moderate-intensity exercise can have a wide range of health benefits, such as reducing inflammation, enhancing immunity, and reducing respiratory viral infections.^[6]^ A study of the early effects of the COVID-19 pandemic on physical activity and sedentary behavior in children living in the USA has shown that pandemic prevention measures have led to a decrease in physical activity and a significant increase in sedentary time among American children, with the negative effect of individuals’ weakened immunity and increased vulnerability to the virus.^[7]^

Bibliometric analysis is a method of statistical analysis of the research results. This research method has been applied in several fields. Through the analysis of authors, institutions, keywords, countries, cited authors, cited journals, etc., it provides users with research information and ideas. Researchers have a clear understanding of the past and present of the field, which helps them master hot issues and cutting-edge trends in the field.^[8]^ While there were publications on COVID-19 and physical activity, including meta-analyses, to our knowledge, none of these publications used visualization methods for analysis. The current bibliometric analysis software includes Citespace^[9]^ (Drexel University, Philadelphia, PA) and VOSviewer^[10]^ (Leiden University, Leiden, Netherlands). We want to investigate recent hot topics and research hotspots in the years to come. Guiding researchers in the field of sports medicine to intervene in changing levels of physical activity in the context of the COVID-19 pandemic, providing people with better exercise guidance and advice, and identifying potential collaborators and collaborating institutions.

## 2. Materials and Methods

### 2.1. Data source and search strategy

**Figure 1.**
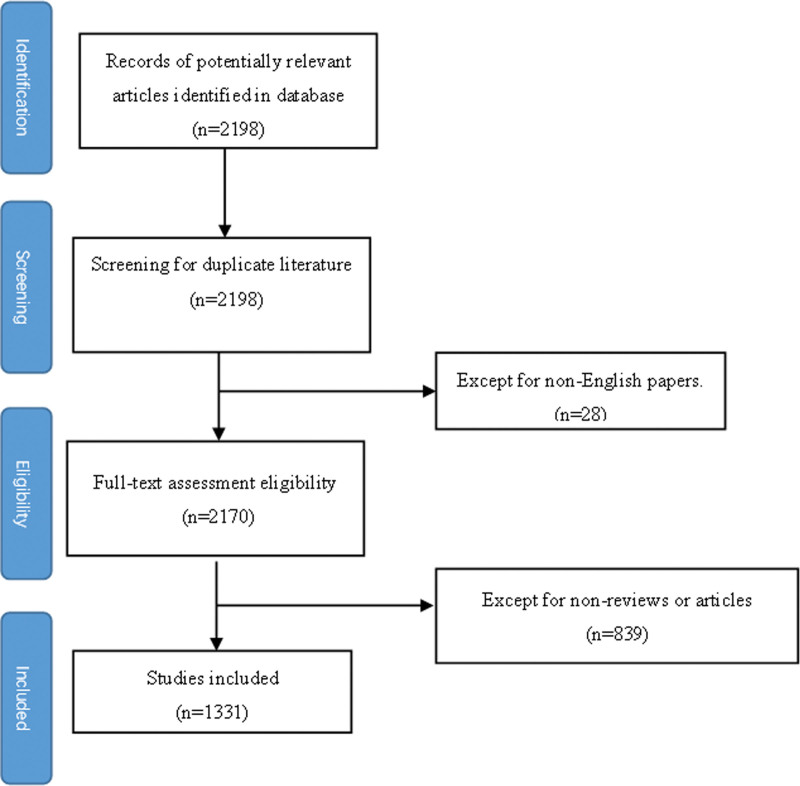
The flow chart.

All publications were searched using the Web of Science core collection database. We also used the Science Citation Index Expanded (SCI-Expanded) database of Web of Science (WOS) as the source of the available databases. We set the period of publication paper retrieval from January 1, 2020, to February 14, 2022. The search strategy was set as the title= (((TI = (exercise* OR kinesitherapy OR training OR “physical activit*” OR sport* OR fitness OR walk* OR run* OR swim* OR jog* OR cycling OR pilates* OR yoga OR qigong OR “tai chi”)) AND TI=(“COVID 19” OR “COVID-19” OR “SARS-CoV-2 Infection*” OR “SARS CoV 2 Infection*” OR “2019 Novel Coronavirus Disease” OR “2019 Novel Coronavirus Infection” OR “2019-nCoV Disease*” OR “2019 nCoV Disease” OR “COVID-19 Virus Infection*” OR “COVID 19 Virus Infection” OR “Coronavirus Disease 2019” OR “Coronavirus Disease-19” OR “Coronavirus Disease 19” OR “Coronavirus 2 Infection” OR “SARS Coronavirus 2 Infection” OR “COVID-19 Virus Disease*” OR “COVID 19 Virus Disease” OR “2019-nCoV Infection*” OR “2019 nCoV Infection” OR “COVID-19 Pandemic*” OR “COVID 19 Pandemic”)) AND DT = (Article OR Review)) AND LA = (English). The inclusion criteria are shown in Figure 1. The language was restricted to English, and 28 non-English papers were excluded. Document types were limited to articles and reviews, excluding letters, meeting abstracts, published editorials, materials, book reviews, conference presentations, news items, and corrections. After excluding 839 studies, 1331 studies were finally included.

### 2.2. Data analysis

CiteSpace 5.8.R3 was used to extract and analyze the number of publications (including outputs, authors, journals, countries, and institutions), citation frequency (including co-cited authors, co-cited journals, and co-cited references), and co-occurring keywords to track research trends and hotspots. The larger the size of the nodes in the map generated by the software, the higher the number of publications or frequency of citations, and the lines between nodes represent cooperative connections. The thicker the lines, the closer the connections. Centrality measures the importance of a node in the overall map-network structure. Centrality > 0.1 is considered an important node. Microsoft Excel 2019 was used to generate line charts of the number of publications and citations. A bibliometric visualization map with a timeline was generated using VOSviewer 1.6.17.

### 2.3. Visual analysis process

The analysis of the diagrams created by the software follows the principles of quantitative before qualitative, holistic before partial and chronological development, in terms of structure, time, and content. The content of the analysis includes: Illustration of the diagram. It explains the properties of the objects represented by the nodes and connecting lines; The overall structure of the diagram and the clustering analysis; and The analysis of high-frequency nodes and high centrality nodes.

### 2.4. Research ethics

The research was conducted as a bibliometric analysis. All data sources were available on the Internet, and no animal or human subjects were involved. Therefore, permission was not required from the ethics committee.

## 3. Results

### 3.1. Analysis of publications outputs and citations

**Figure 2.**
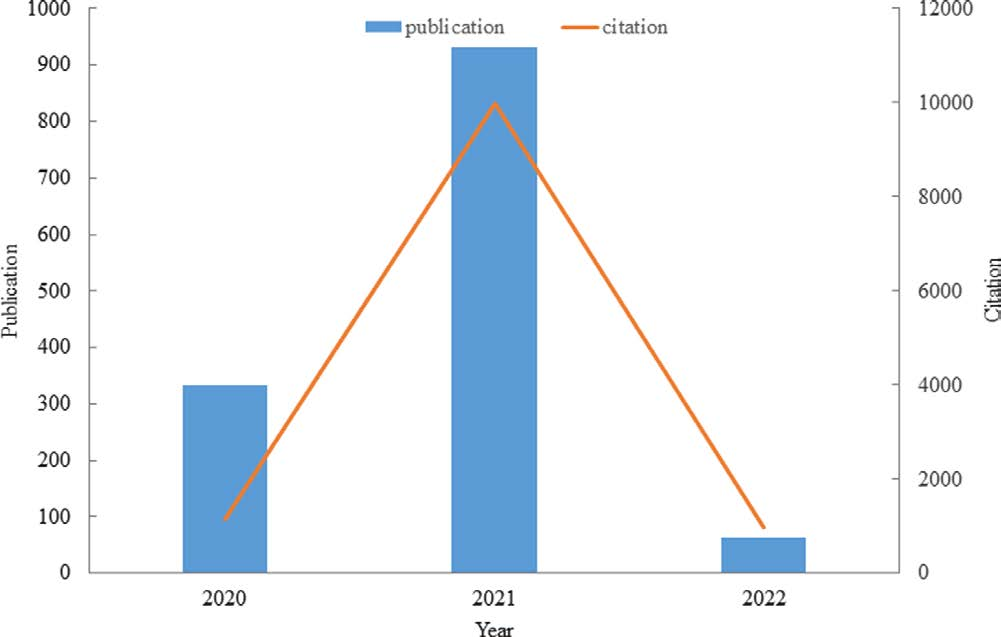
The number of annual publications and annual citations.

Since January 1, 2020, to February 14, 2022, 1331 studies met the inclusion criteria, including 1184 articles and 147 reviews. We imported the data into Microsoft Excel to obtain a histogram of the annual output distribution (Fig. 2). The histogram shows that the number of studies published in 2021 increased from 334 in 2020 to 932 in 2021, and the number of studies published in 2021 was nearly three times that in 2020. The retrieval date was February 14, 2022, and the number of studies published in two months reached 65. We believe that with the development of the epidemic and deepening novel coronavirus research, more novel coronavirus studies will be published in the future.

Figure 2 shows that 1331 studies were cited 12086 times. Through the line chart of annual citations, we found that the studies in 2021 were cited 9975 times, the highest in the previous two years. The number of co-citations in 2020 was 1146, while the number of co-citations in the first two months of 2022 reached 965. Therefore, we estimated that this line chart will show a straight upward trend by the end of 2022.

### 3.2. Analysis of authors and co-cited authors

**Figure 3.**
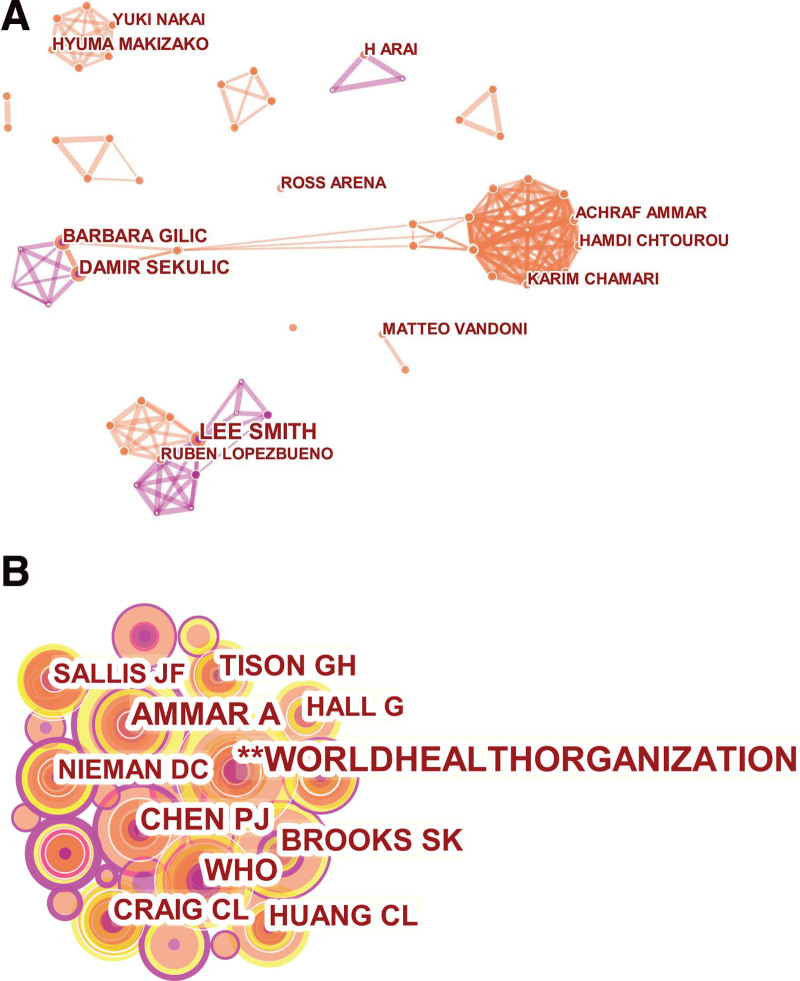
(A) Map of authors of publications. (B) Map of co-cited authors of publications.

Visualizations can provide information about influential authors and potential collaborators and can help researchers build collaborative relationships. From 2020 to 2022, 8548 authors published 1331 studies on COVID-19 and physical activity. To summarize the collaboration between the authors, we created a network map (Fig. 3A). The author’s cooperation map was generated using CiteSpace with 158 nodes and 360 links (Fig. 3A). The network density was 0.029. The top 10 active authors (Table 1) who has published articles related to COVID-19 and physical activity were SMITH L, SEKULIC, GILIC B, MAKIZAKO H, NAKAI Y, CHAMARI K, ARAI H, LOPEZ-BUENO R, AMMAR A, and VANDONI M. SMITH L ranked first with 11 publications, followed by SEKULIC D (8 publications). This figure shows that there are three main research teams in this field. The team represented by SMITH L^[11–14]^ focused on changes in adults’ physical activity levels and mental health during COVID-19 confinement, whereas SEKULIC D’s team^[15,16]^ focused on the effects of different environmental factors on adolescents’ physical activity during confinement. MAKIZAKO H’s team^[17–21]^ studied changes in physical activity, pain management, sleep quality, and health in older people affected by the pandemic. At the same time, the author’s collaboration network diagram also shows that there was little collaboration between different research teams due to differences in geography and institutions.

**Table 1:** Top 10 active authors, co-cited authors.

Rank	Author	Published article	Co-cited author	Cited times	Co-cited author	Centrality
1	SMITH L	11	**WORLDHEALTHORGANIZATION	214	DING D	0.43
2	SEKULIC D	8	AMMAR A	135	NIEMAN DC	0.37
3	GILIC B	7	WHO	115	BULL FC	0.37
4	MAKIZAKO H	6	BROOKS SK	110	XIANG M	0.28
5	NAKAI Y	5	CHEN PJ	103	DUNTON GF	0.26
6	CHAMARI K	5	CRAIG CL	79	CHEN PJ	0.25
7	ARAI H	5	HUANG CL	78	DWYER MJ	0.25
8	LOPEZ-BUENO R	5	TISON GH	76	AMMAR A	0.24
9	AMMAR A	5	NIEMAN DC	68	DUNCAN GE	0.24
10	VANDONI M	5	HALL G	68	KNELL G	0.21

A total of 1331 papers was published, and 484 authors were cited. Pruning the map using Pathfinder results in a simplified network structure diagram. There were 484 nodes and 576 connecting lines (Fig. 3B). By hiding anonymous authors, we can see that the top 5 authors cited were: **WORLDHEALTHORGANIZATION (214 times), Ammar A (135 times), WHO (115 times), Brooks SK (110 times), and CHEN PJ (103 times). The high number of co-citations indicates that the work of these authors also contributed to the development of related disciplines. Among all co-cited authors, DING D had the highest centrality (0.43), followed by Nieman DC and Bull FC (0.37). Based on the number of co-citations and centrality analysis, AMMAR A of Germany, CHEN PJ of Taiwan and China, and NIEMAN DC of the USA were the core researchers in this field. Their research focuses on life sciences and sports medicine, and their research has had a significant impact in this field.

### 3.3. Distribution of journals and co-cited journals

**Figure 4.**
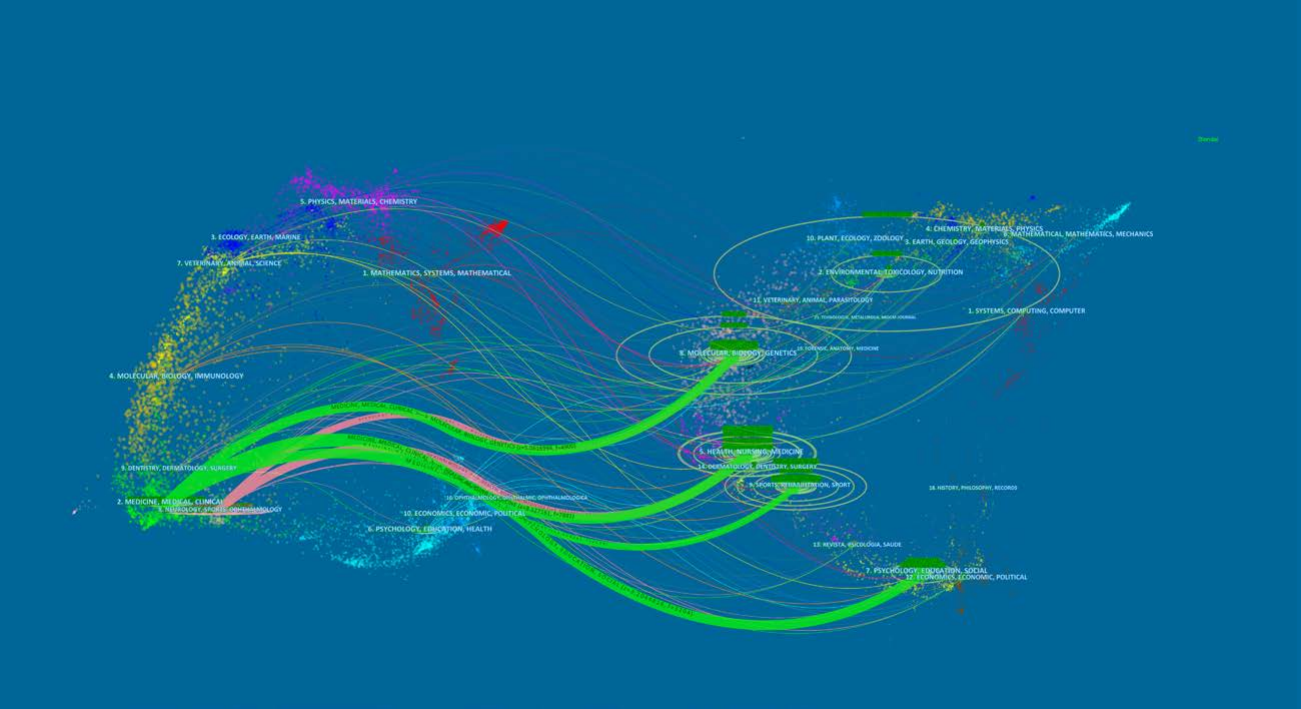
The dual-map overlay of journals.

**Figure 5.**
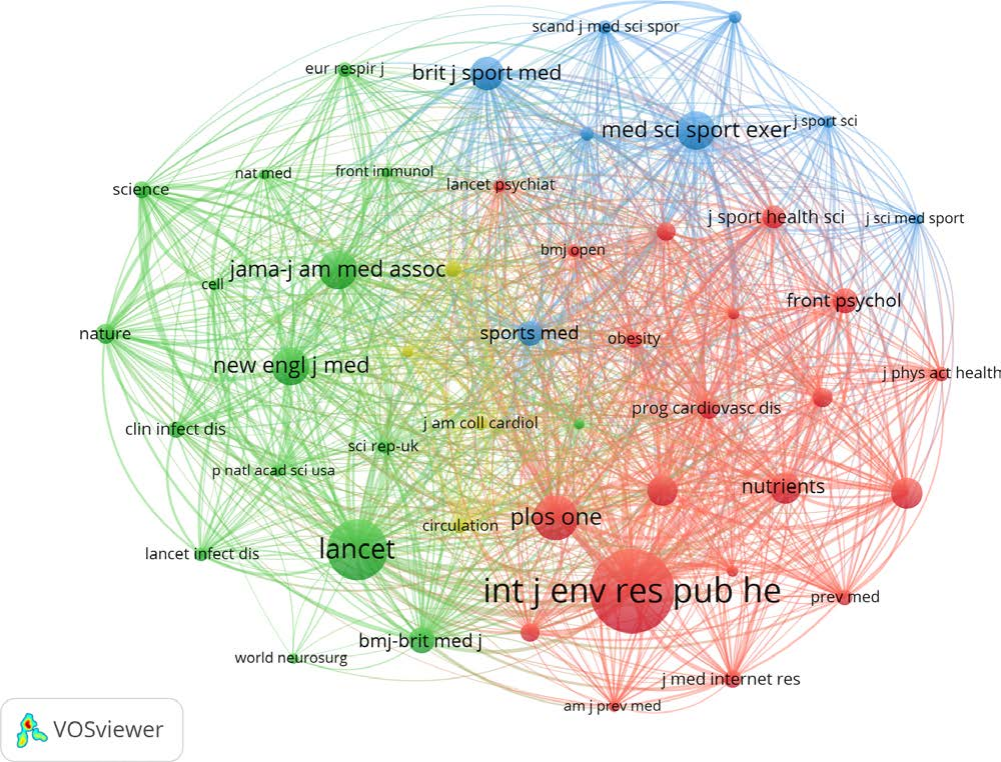
A map of co-cited journals of publications.

The nodes in the map represent journals and the lines between nodes represent relationships. The greater the node area, the more co-citations that occur. The purple rings represent centrality, and the nodes with high centrality are key points on the map. The top 10 journals that published research papers related to COVID-19 and physical activity are shown in Table 2. Among these, *International Journal of Environmental Research and Public Health* was the most productive, with 179 publications. Figure 4 shows a dual map obtained using CiteSpace. The citing journals are on the left side of the dual-map, the cited journals are on the right side, and the lines on both sides represent the citation relationship. It can be seen from the figure that a large number of papers were published in *“neurology, sports, ophthalmology”* journals, most of which cited journals in the fields of *“health, nursing, medicine”* journals. Figure 5 and Table 3 show the co-cited times and centralities. Lancet had the highest co-cited count (527) and *International Journal of Clinical Practice* had the highest centrality (0.09). Based on a comprehensive analysis of journal citations and centrality, *Lancet* (IF = 79.323), *International Journal of Environmental Research and Public Health* (IF = 3.39), and *International Journal of Behavioral Nutrition and Physical Activity* (IF = 6.457) had the value of quickly understood basic principles and tracked trends.

**Table 2:** Top 10 academic journals.

Rank	Publications	Journal	IF (2020)
1	179	International Journal of Environmental Research and Public Health	3.39
2	44	Sustainability	3.251
3	27	Medicine	1.889
4	23	BMC Public Health	3.295
5	22	Nutrients	5.719
6	19	Frontiers in Public Health	3.709
7	19	PLoS One	3.24
8	16	BMC Medical Education	2.463
9	16	Healthcare	2.645
10	12	BMJ Open	2.692

**Table 3:** Top 10 co-cited journals.

Rank	Co-cited counts	Cited journal	Centrality	Cited journal
1	527	Lancet	0.09	International Journal of Clinical Practice
2	497	International Journal of Environmental Research and Public Health	0.08	Gastrointestinal Endoscopy
3	410	PLoS One	0.07	International Journal of Environmental Research and Public Health
4	336	JAMA-Journal of the American Medicine Association	0.06	International Journal of Behavioral Nutrition and Physical Activity
5	292	New England Journal of Medicine	0.06	Preventive Medicine
6	291	Medicine and Science in Sports and Exercise	0.06	Lancet Infectious Diseases
7	263	BMC Public Health	0.06	Aging Clinical and Experimental Research
8	260	British Journal of Sports Medicine	0.05	Lancet
9	248	British Medical Journal	0.05	Nutrients
10	229	International Journal of Behavioral Nutrition and Physical Activity	0.05	European Respiratory Journal

### 3.4. Analysis of co-cited references

A total of 38266 references was generated from the 1331 records. The study period was from 2020 to 2022, and the time slice was 1. Table 4 lists the top five co-cited references for counts and Table 5 lists the top five co-cited references for centrality.

**Table 4:** Top 5 co-cited references in terms of co-citation counts.

Rank	Co-citation counts	Cited reference	Representative author (publication year)
1	117	Effects of COVID-19 Home Confinement on Eating Behaviour and Physical Activity: Results of the ECLB-COVID19 International Online Survey	Ammar A (2020)
2	98	Coronavirus disease (COVID-19): The need to maintain regular physical activity while taking precautions	Chen PJ (2020)
3	90	The psychological impact of quarantine and how to reduce it: a rapid review of the evidence.	Brooks SK (2020)
4	76	Worldwide Effect of COVID-19 on Physical Activity: A Descriptive Study	Tison GH (2020)
5	64	The Impact of COVID-19 on Physical Activity Behavior and Well-Being of Canadians	Lesser IA (2020)

**Table 5:** Top 5 co-cited references in terms of centrality.

Rank	Centrality	Cited reference	Representative author (publication year)
1	0.11	Coronavirus disease (COVID-19): The need to maintain regular physical activity while taking precautions	Chen PJ (2020)
2	0.10	Effects of COVID-19 Home Confinement on Eating Behavior and Physical Activity: Results of the ECLB-COVID19 International Online Survey	Ammar A (2020)
3	0.10	Mental Health and the COVID-19 Pandemic	Pfefferbaum B (2020)
4	0.09	Clinical Characteristics of Covid-19 in China	Zavascki AP (2020)
5	0.09	Impact of the COVID-19 pandemic on urology residency training in Italy	Amparore D (2020)

The article with the highest co-cited counts (Table 4), published in 2020, was found by Ammar et al^[22]^ (counts were 117) that home quarantine measures were necessary to protect public health during the COVID-19 pandemic, but most people exhibit decreased levels of physical activity and unhealthy eating patterns that lead to an increased risk of disease and death. The researchers also suggested that using ICT to support exercise at home, maintain healthy eating habits, and add beneficial social factors are important for maintaining health. The article with the highest centrality (Table 5) was published by Chen PJ et al^[23]^ in 2020 (with a centrality of 0.11), indicating that long-term family confinement would change people’s lifestyles and lead to psychological problems such as anxiety and depression.

As can be seen from the co-cited references in Tables 4 and 5, the level of physical activity of the public decreased to varying degrees during the COVID-19 pandemic. Therefore, maintaining physical activity through various means is of great significance in maintaining physical health.

### 3.5. Analysis of countries and institutions

**Figure 6.**
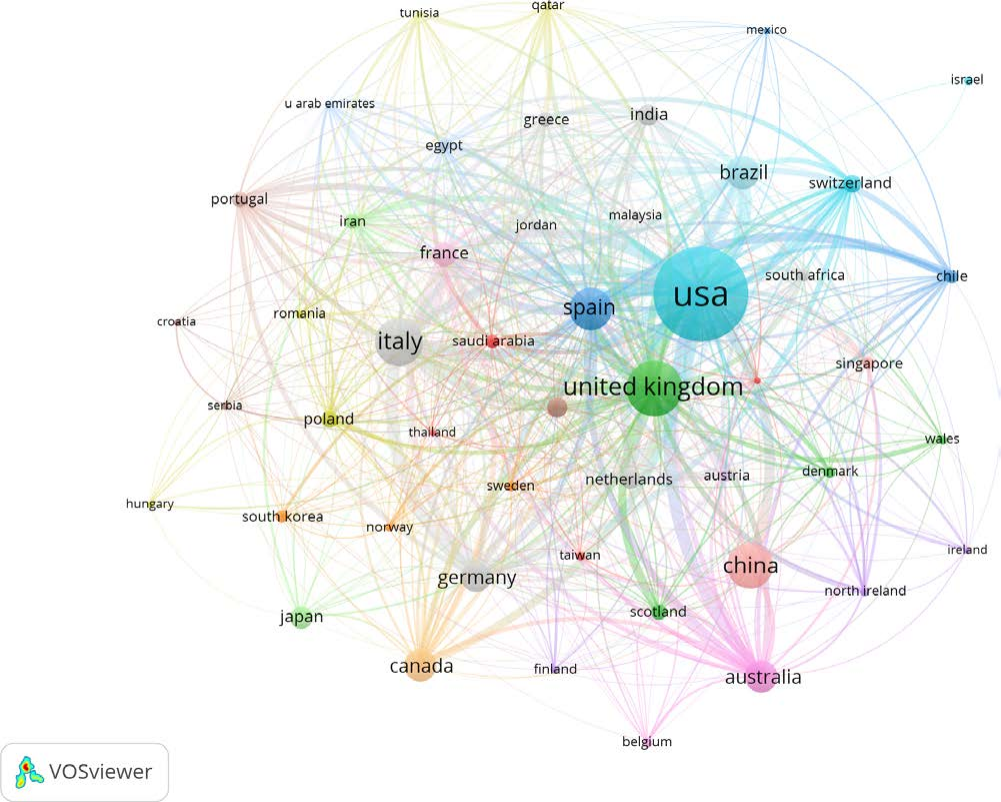
A map of countries.

The map of the country was generated using VOSviewer. The 1331 studies were published in 110 countries. For a better visualization, we selected 49 countries with more than 10 studies each. The top five countries (Table 6) with the most published papers was the USA (344), followed by the United Kingdom (168), Italy (137), China (130), and Spain (117). Of the top five countries, only China is a developing country. It can be seen that developed countries are devoting more resources to this area. However, since the epidemic was first reported in China, the Chinese government has mobilized a large amount of human and material resources to prevent its spread, which has also provided a reference for global epidemic prevention and control. Figure 6 shows the connections between countries. There is a cooperative relationship between the countries. We used CiteSpace to calculate the centrality of each country. Centrality means that a node establishes a bridge between two unrelated nodes. A high intermediate centrality convex indicates the importance of the nodes in the structure. In addition, the United Arab Emirates ranked first with a centrality of 0.69, followed by Oman and Iran.

**Table 6:** Top 10 countries.

Rank	Country	Publication	Country	Centrality
1	USA	344	U ARAB EMIRATES	0.69
2	UNITED KINGDOM	168	OMAN	0.28
3	ITALY	137	IRAN	0.27
4	CHINA	130	GERMANY	0.23
5	SPAIN	117	CHILE	0.22
6	BRAZIL	86	LIBYA	0.22
7	CANADA	82	YEMEN	0.21
8	AUSTRALIA	81	MEXICO	0.20
9	GERMANY	74	PERU	0.19
10	FRANCE	56	SINGAPORE	0.17

**Figure 7.**
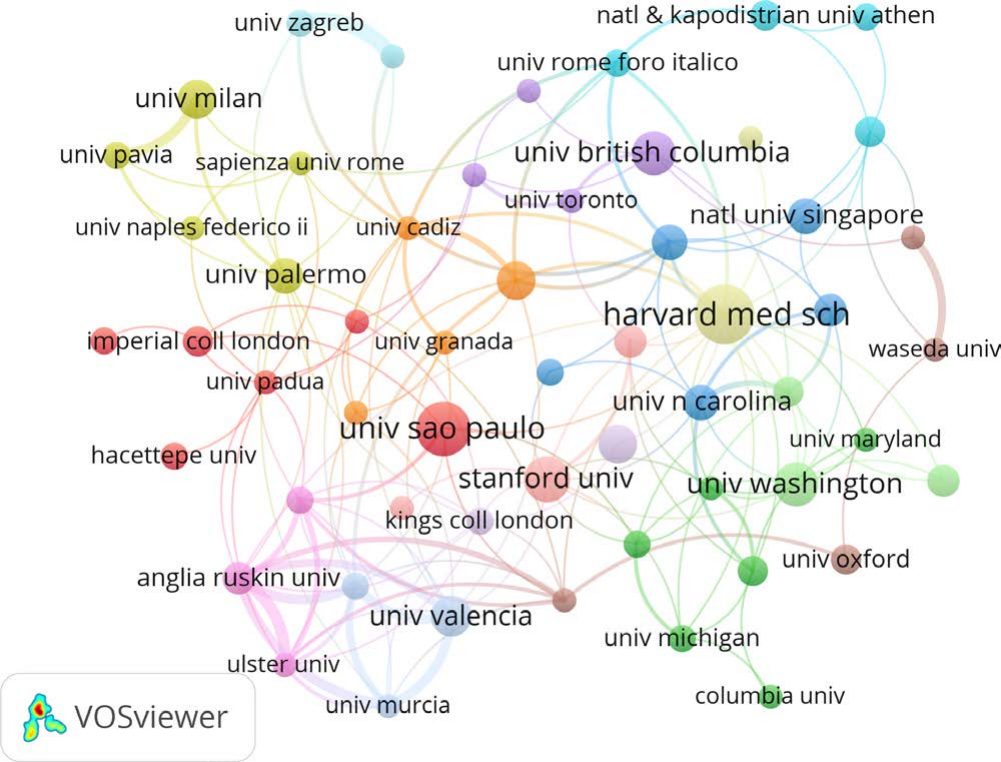
A map of institutions.

**Figure 8.**
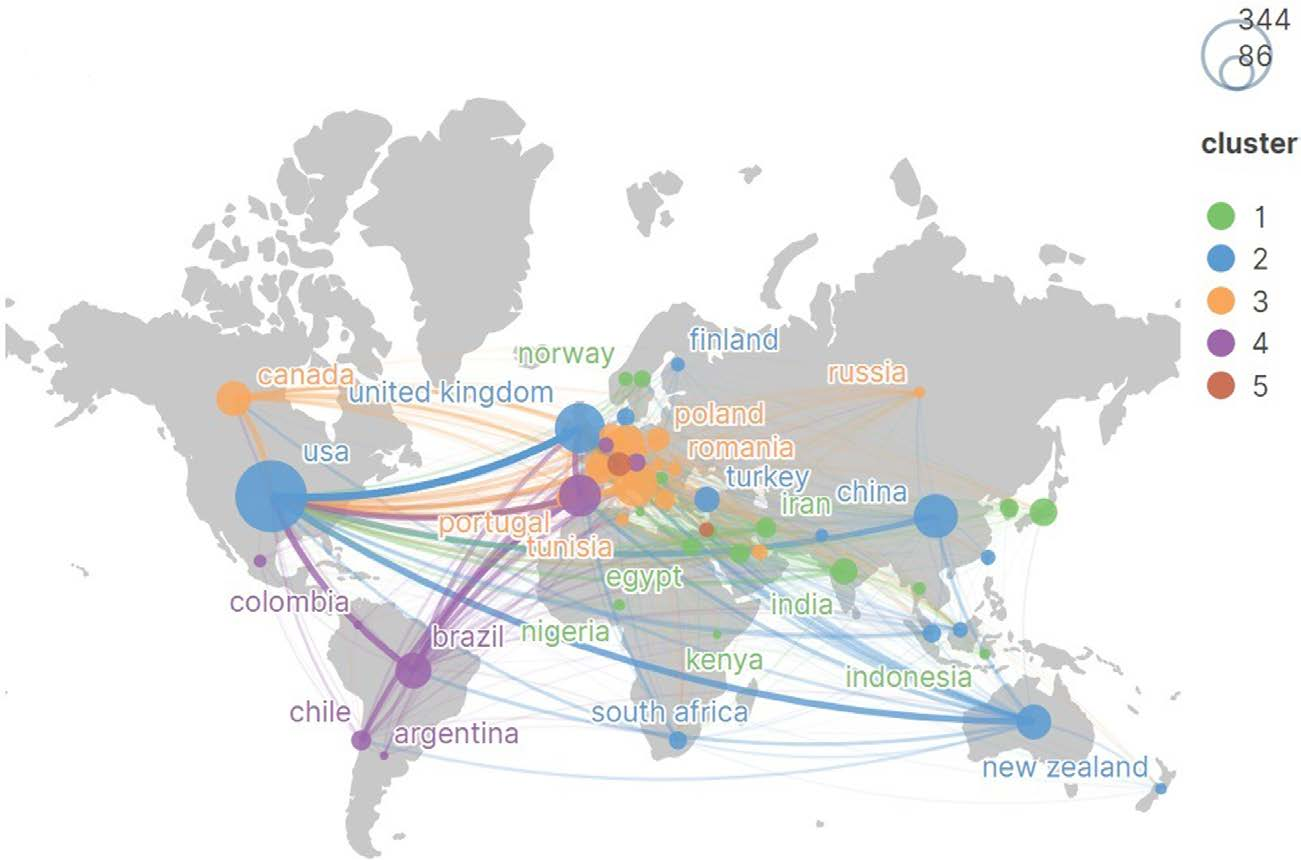
The distribution of countries/region.

We used VOSviewer to obtain a collaborative map of the institutions (Fig. 7). Totally, 2881 institutions made contributions to COVID-19 and physical activity. To obtain a better visualization, we included 57 institutions that had published at least eight times. The top five institutions are Harvard Medical School (21), the University of São Paulo (19), Stanford University (16), the University of Washington (15), and the University of British Columbia. Table 7 shows the top 10 institutions in terms of several publications. Similarly, we used CiteSpace to analyze the centrality of the institution. Combined with publications (21) and centrality (0.36), Harvard Medical School contributed the most in this area. As the coronavirus pandemic has spread across the globe, countries have stepped up vaccine development and have become more connected to each other. Countries and regions are presented on the world map in Figure 8.

**Table 7:** Top 10 institutions.

Rank	Institution	Publication	Institution	Centrality
1	Harvard Med Sch	21	Harvard Med Sch	0.36
2	Univ Sao Paulo	19	Emory Univ	0.33
3	Stanford Univ	16	Univ Basel	0.31
4	Univ Washington	15	Univ Politecn Madrid	0.30
5	Univ British Columbia	15	Univ Jyvaskyla	0.26
6	Univ Valencia	14	Loughborough Univ	0.24
7	Univ Politecn Madrid	13	Univ Rome Foro Italico	0.22
8	Univ Milan	13	Univ Colorado	0.21
9	UCL	13	Boston Childrens Hosp	0.21
10	Univ Queensland	12	Univ Queensland	0.20

As COVID-19 spread worldwide, the USA and Harvard Medical School were at the center of high-level collaboration with countries and institutions. Collaboration helped researchers share resources and exchange experiences, which were critical for the further development of COVID-19 and physical activity research.

### 3.6. Analysis of co-occurring keywords

**Figure 9.**
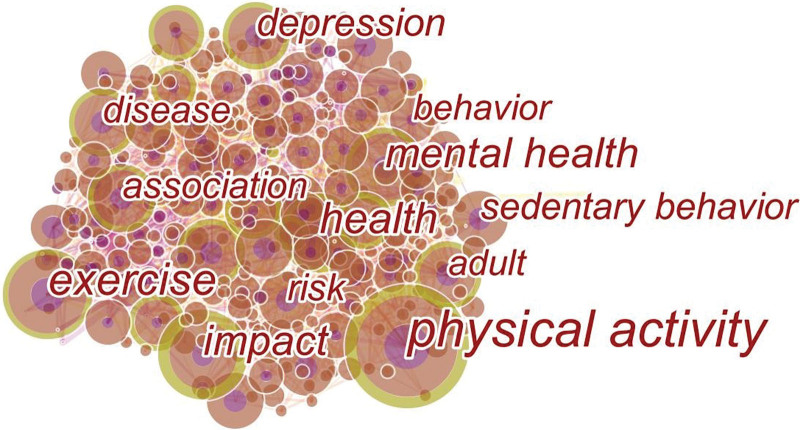
A map of co-occurring keywords.

The co-occurrence keyword map reflects the prevalence of COVID-19 research and its physical activity. Nodes represent keywords and lines between nodes represent co-occurrence relationships. The larger the node area, the higher the frequency. Nodes with high centrality were the focus of publications. The map was constructed with 336 nodes and 1365 links (Fig. 9). According to the analysis of the frequency and center of comorbidity (Table 8; Fig. 9), the frequency of “physical activity” was the highest (330 times), and the center of “fatigue” was the highest (0.10), indicating that since COVID-19 swept the world, the level of physical activity of the public had a greater impact, and the public attached more importance to physical exercise. “Fatigue” refers to fatigue as a major symptom of the COVID-19 pandemic or physical and mental fatigue among members of the public who are not sick due to social restrictions and decreased levels of physical activity.

**Table 8:** Top 10 co-occurring keywords.

Rank	Frequency	Keyword	Centrality	Keyword
1	330	physical activity	0.10	fatigue
2	134	exercise	0.08	physical activity
3	115	health	0.07	association
4	103	mental health	0.07	acute respiratory syndrome
5	92	impact	0.06	impact
6	69	depression	0.06	quality of life
7	63	sedentary behavior	0.06	obesity
8	61	association	0.05	risk
9	56	disease	0.05	adult
10	55	risk	0.05	performance

## 4. Discussion

This study used CiteSpace and VOSviewer to perform a bibliometric analysis of papers published on COVID-19 and physical activity research between January 1, 2020, and February 14, 2022. This study reviewed the research situation, COVID-19 hotspots, and physical activity.

### 4.1. COVID-19 and physical activity: general information and global trends

The study evaluated two paper types: articles (1184) and reviews (147). Three times as many studies have been published in 2021 as of 2020, mainly related to the explosive growth of COVID-19 in a short period. The downward trend in the number of studies published in 2022 was related to limited time, as the number of studies published in 2022 reached 65 in just two months. More articles will be published in the future as vast medical resources and scientific research are being poured into the COVID-19 outbreak around the world.

The top five authors were SMITH L of Anglia Ruskin University, SEKULIC D and GILIC B of the University of Split, MAKIZAKO H of Kagoshima University, and NAKAI Y of Daiichi Institute of Technology. Most of them were from sports science or biomedical backgrounds. The results showed that the top 10 journals (Table 2) accounted for 28% of the total. *International Journal of Environmental Research and Public Health* published 179 papers (13.45%), The second was *SUSTAINABILITY* (44 publications, 3.31%), so related articles were mainly published in these two journals. *International Journal of Environmental Research and Public Health* had been identified as the core journal in the field of COVID-19 and physical activity through a comprehensive analysis of published papers, several published papers, and centrality, and its published papers reflected the basic theories of the research field.

The country that published the most papers in this field was the USA, and the USA was the central partner of other countries, which was probably related to the fact that the USA was a country with medical technology and rich scientific research resources. However, when COVID-19 broke out globally, the USA had some of the highest diagnosis and death rates in the world over a long time owing to political, cultural, and social factors, forcing researchers to delve into how to increase physical activity levels to stay healthy during the COVID-19 pandemic. The COVID-19 outbreak was first reported in Wuhan, China. China was also the first country to adopt a lockdown policy, which quickly controlled the infection and death rate of the virus and achieved a phased victory, which also provided a reference direction for other countries to make public health decisions. For example, under the confinement policy, there are potential health problems. To cope with the unprecedented limits on outdoor sports, China actively promoted healthy, uninfected disease exercises that occupied the home. In addition, because of the COVID-19 patients, treatment guidelines in the rehabilitation program of a combination of Chinese and Western medicine diagnosis and treatment schemes have been proposed. Among them, the traditional Chinese exercise therapy intervention,^[24]^such as taiji,^[25]^ Qigong,^[26]^ Baduanjin,^[27]^ and the comprehensive intervention of traditional Chinese medicine, played a significant role. In the future, researchers should pay more attention to potential health problems associated with major public health events and coordinate the formulation of exercise guidelines and prescriptions for related diseases, which may be more conducive to the development of exercise therapy. More importantly, cooperation between experts from other professional backgrounds should be strengthened.

### 4.2. COVID-19 and physical activity: research hotspots

This study investigated the current hot spots of COVID-19 and physical activity research in terms of co-cited authors, co-cited keywords, and co-cited references, helping researchers to explore the distribution of topics within specific disciplines.

Based on the analysis of the number of co-citations and centrality, the results of AMMAR A, CHEN PJ, and NIEMAN DC were as follows: AMMAR A’s team^[22,28–31]^ conducted multiple, multicenter cross-sectional studies showing that quarantine policies during COVID-19 can negatively impact health by changing people’s activity levels and dietary patterns.^[22]^ Decreased exercise can also lead to decreased sleep quality, depression, anxiety, and other mental health problems.^[32]^ CHEN PJ’s^[23]^ team proposed in detail the exercise methods and requirements for family exercise, such as stair climbing, sit-ups, yoga,^[33]^ and qigong training. Some exercises were better than none, and more exercises were better than a little. Appropriate intensity and duration of exercise should be maintained every day^[34]^ and at least 30 minutes of moderate exercise should be combined with at least 20 minutes of vigorous exercise. NIEMAN DC et al^[35]^ have shown that, driven by the pandemic, most sports medicine has shifted to telemedicine, providing online advice on the best training programs and physical activities for those in need. This coincides with AMMAR A recommendation to promote ICT technologies such as home sports games and online fitness software.

According to co-cited references, social distancing measures advocated by governments to curb the spread of the disease since the COVID-19 outbreak have been associated with decreased physical activity and negative psychological effects. Tison et al^[36]^ study found that through a large-scale sampling of people’s daily steps, the total number of global steps declined significantly, indicating that the social distancing policy has an impact on overall physical activity. Brooks et al^[37]^ research showed that the psychological impact of isolation is extensive and substantial, and may be long-term. Therefore, the state should provide the public with psychosocial support beyond medical services, and the psychosocial problems of medical workers should not be ignored either.^[38]^ In addition, public health emergencies affect public sentiment, and gender differences exist. Changes in physical activity have a greater impact on women’s mental states than on men’s.^[39]^ To reduce mental stress, we must promote environmental opportunities and physical activity support for vulnerable groups, especially women and older adult. In addition to incorporating physical activity into a lifestyle,^[40]^ the use of digital technology^[41]^ also contributes to reducing psychosocial stress associated with family isolation.

Our keyword co-occurrence analysis revealed that the four most commonly used keywords were physical activity, exercise, health, and mental health. The physical activity level was the best predictor of mental health. Appropriate physical activity, depending on the intensity, duration, and type of exercise, can strengthen the immune system and reduce inflammation and disease rates in individuals of all ages.^[11,42,43]^ Reduced exercise levels lead to reduced endorphin release,^[44]^ and exercise levels are also associated with the regulation of circulating Neuro-nutrients.^[45]^ Therefore, maintaining the continuity of exercise training is of great significance for maintaining a nutritional level, enhancing physique, and reducing mental health problems such as depression and anxiety.^[45–48]^

Confinement policies limit public physical activity, and this inactive lifestyle suppresses the immune response, increases the risk of viral infection, and worsens the health of many people who are forced to self-isolate without contracting the virus. Therefore, it is important to scientifically maintain levels and patterns of exercise at home. The World Health Organization recommends 150 minutes of moderate exercise or 75 minutes of vigorous exercise per week, preferably in combination.^[49]^ Electronic information technology^[41]^ to take online fitness classes at the right time, such as low - and moderate-intensity resistance exercises,^[50]^ reduce sedentary time, make exercise more interactive and fun, and help the body relax through post-exercise meditation and deep breathing.^[51]^ Therefore, it is particularly important during pandemic isolation to encourage home-based exercise training programs for healthy people forced to stay at home or for those with chronic diseases.^[40]^

Exercise therapy has been increasingly recognized and accepted during the COVID-19 pandemic. Well-designed, high-quality research results will also be progressively published in high-quality journals, which will help spread research on COVID-19 and physical activity, as well as inform public health decisions.

### 4.3. Study strengths and limitations.

This study is the first bibliometric analysis to assess trends in COVID-19 and physical activity research in the WOS Science Extended Science Citation Index over three years following the COVID-19 outbreak. The 1331 papers retrieved from the study were published in 558 different academic journals, not limited to a single academic journal, and yielded a wealth of data. In addition, the study of literature metrology analysis, including the values of the annual output and cited, countries, institutions, periodicals’ distribution, authors, and references, as well as the co-occurrence of keywords, cited authors, cited journals, and cited literature analysis, can promote the understanding of the academic development of a specific subject and also help to determine the theme of the emerging and development direction in the future.

This study had some limitations. First, we limited the data source to SCI-expansion of WOS, excluding PubMed and other databases, which may have resulted in incomplete real data. Therefore, some of the data and analyses in this study had some deviations. Second, bibliometrics cannot effectively consider the validity and scientific rigor of the publications. Highly cited publications do not necessarily have a high scientific quality. Despite these limitations, we believe that our findings are a valid representation of global research in the field of COVID-19 and physical activity.

## 5. Conclusion

In summary, this study provides valuable information for nearly three years of research on COVID-19 and physical activity. The current state of research on the changes and the effects of physical activity during COVID-19 confinement suggests that research on the treatment and prevention of COVID-19 through exercise interventions has great potential for development and needs to be strengthened collaboration with other countries. The study shows that research on COVID-19 and physical activity is becoming more extensive globally between 2020 and 2022, indicating that this field of research is well-developed and promising. The USA, England, Italy, and China are core research forces. Taken together, this study provides a historical perspective on COVID-19 and physical activity research that helps us understand leading research countries and institutions, core journals, overall trends, hotspots, and research frontiers.

## Acknowledgments

The authors would like to express their appreciation for Cite Space, the free software developed by CM Chen, and VOSviewer, the free Java-based software developed by Van Eck and Waltman.

## Author contributions

**Conceptualization:** Xihua Fu.

**Data curation:** Chunlong Liu, Zhijie Zhang.

**Formal analysis:** Yuting Zhang, Mengtong Chen.

**Funding acquisition:** Xihua Fu.

**Visualization:** Yuting Zhang, Mengtong Chen.

**Writing – original draft:** Yuting Zhang, Mengtong Chen.

**Writing – review & editing:** Chunlong Liu, Zhijie Zhang.
